# Relative Effect of a Common Environment upon Enamel

**Published:** 1901-05

**Authors:** Frederick L. Bogue

**Affiliations:** New York


### International Dental Journal.

Vol. XXII. May, 1901. No. 5.

### Original Communications.[1]

### RELATIVE EFFECT OF A COMMON ENVIRONMENT UPON ENAMEL.[2]

BY FREDERICK L. BOGUE, M.D., D.D.S., NEW YORK.

Mr. President and Gentlemen,—The subject of the short paper which I have the honor of presenting to you this evening is "Relative Effect of a Common Environment upon Enamel."

Dr. Black, in his paper upon "The Physical Characters of the Human Teeth," confined his experiments and derived his conclusions from a study of the dentine. As teeth are more or less perfectly covered with enamel, and until this is penetrated the dentine is not attacked, it seemed fitting that some experiments should be made upon this important structure to ascertain if it was responsible for the apparent differences between Dr. Black's conclusions and those held by the profession up to that time, which were based principally upon more or less careful clinical observation.

That environment has a greater bearing on the liability to decay, and that the physical and chemical properties of the teeth of dif-

[1] The editor and publishers are not responsible for the views of authors of papers published in this department, nor for any claim to novelty, or otherwise, that may be made by them. No papers will be received for this department that have appeared in any other journal published in the country.

[2] Read before The New York Institute of Stomatology, February 5, 1901.

ferent individuals are more nearly alike than had ever been appreciated before, I think is perfectly true, but that the rapidity of decay is entirely dependent upon the environment, and that the physical and chemical structure of the teeth has no bearing, I think has not been adequately proved.

Clinically we sometimes discover a well-defined cavity in the approximal surface of a tooth, while the adjoining tooth is in perfect condition. These teeth have been exposed to the same environment; how do we account for the greater resistance shown by one of the teeth? If teeth in the same mouth show differences in the amount of resistance, is it not natural to suppose that there may be even greater differences in teeth taken from various individuals?

There are also deviations from the normal forms of teeth, due to interrupted development, sequelæ of some special disease, such as measles or syphilis. Are teeth of that character, aside from their defective mechanical structure, no more prone to decalcification than perfectly normal teeth?

According to Mr. Tomes, the microscope shows that "the tissues are not only deficient in quantity, but they are defective also in quality. The elements of the enamel are imperfectly combined, hence the tissue is porous, yellow, opaque, and very fragile." If it were possible to have a common environment and teeth of exactly the same chemical composition, there would still be differences in the degree of disintegration depending upon what we term predisposing causes,—*i.e.*, 1, regularity or irregularity of the teeth; 2, differences in individual shape of the teeth and their location as regards the salivary ducts; 3, variations in the thickness of the enamel-cap, and differences in its mechanical structure, whether without blemish and fused at the bottom of the sulci or, on the other hand, having pits and grooves in the surface and defective sulci extending entirely through the enamel into the dentine.

These predisposing causes naturally should be eliminated so far as possible in experiments which are intended to show the comparative resistance of different specimens of enamel in the presence of a common decalcifying agent. To accomplish this the teeth were covered with wax except for that portion of the surface which was to be exposed to the action of the solution.

The experiment tried was the production of artificial decay on a number of teeth so prepared, in a mixture of bread and saliva, to ascertain if the rate of decalcification was uniform in the different specimens. The mixture of bread and saliva was the only medium used in the experiments reported this evening, and was chosen first as being the least unpleasant. During the process the teeth were kept at the temperature of the body in a small water oven, the gas flame warming it being controlled by a thermostat.

The first time, seven bottles of the solution, each containing ten teeth, were kept in the oven for two months. As often as was necessary to keep them well covered more of the solution was added. At the end of this time the teeth were removed and carefully examined, and it was found that there were differences in the amount of decalcification. After finding these variations it was thought possible that the environment might have been different in the several bottles, so it was decided to repeat the experiment, placing the teeth in a single vessel. Also, as further investigations were to be made, it was necessary to examine them to determine their condition,—whether the pulp was alive or dead, whether they were decayed or sound, etc.,—and to number them so that they might be kept track of during the work.

The second time one hundred teeth were used and treated in the same way as the first, except that they were all placed in one vessel. At the end of two months these were removed and examined, and again differences were found in the amount of decalcification.

I will pass around a few specimens of the teeth so treated, that the gentlemen may see the difference in the amount of decalcification. These are the extremes, and for this reason are in a measure misleading, inasmuch as the majority of the teeth range between these points, and the degree of difference in them when taken as a whole is much less marked than this.

To determine more accurately the relative degree of decalcification in the different specimens, fifty blocks of enamel were cut from them and the edges filed down until they were all of equal weight (twenty milligrammes). These were exposed to the action of the solution for six weeks. They were then removed and reweighed with the following results: nine weighed eight to eight and one-half miligrammes each; eight, eight and one-half to nine and one-half; sixteen, nine and one-half to ten and one-half; nine, ten and one-half to eleven and one-half; six, eleven and one-half to twelve and one-half. In other words, nine specimens lost between eleven and one-half and twelve milligrammes in weight; eight lost between eleven and one-half and ten and one-half; sixteen lost between ten and one-half and nine and one-half; nine lost between nine and one-half and eight and one-half; and six lost between eight and one-half and seven and one-half milligrammes.

Twenty-five more specimens were cut, each weighing fourteen milligrammes, and were exposed to the solution for two weeks, with the following results: two weighed 13.9 milligrammes each; five weighed thirteen milligrammes each; three weighed 12.9 milligrammes each; three weighed 12.1 milligrammes each; six weighed twelve milligrammes each; three weighed 11.6 milligrammes each; three weighed 11.2 milligrammes each.

The relative specific gravity of the enamel of these teeth was determined to see if there was any relation between the density of the enamel and its liability to decalcification. While the differences in the relative density were rather greater than those found by Dr. Black in his investigations on the dentine, the variations found do not seem to have any fixed relation to the amount of decalcification so far as I can see at present.

Of course, these results are by no means conclusive, as but a single environment was used throughout, and until sound portions of these teeth have been exposed to other environment and differences shown in every instance, it cannot be stated positively that factors other than environment have a bearing on the rapidity of decalcification, but I think it may reasonably be said that the question is an open one.

